# Bilinguals' Plausibility Judgments for Phrases with a Literal vs. Non-literal Meaning: The Influence of Language Brokering Experience

**DOI:** 10.3389/fpsyg.2017.01661

**Published:** 2017-09-25

**Authors:** Belem G. López, Jyotsna Vaid, Sümeyra Tosun, Chaitra Rao

**Affiliations:** ^1^LLAMA Lab, Department of Mexican American and Latina/o Studies, University of Texas at Austin Austin, TX, United States; ^2^Language and Cognition Laboratory, Department of Psychology, Texas A&M University College Station, TX, United States; ^3^Deparment of Psychology, University of Pretoria Pretoria, South Africa; ^4^National Brain Research Center Manasar, India

**Keywords:** language brokering, bilingualism, plausibility, sense-making, literal meaning, figurative meaning

## Abstract

Previous work has shown that prior experience in language brokering (informal translation) may facilitate the processing of meaning within and across language boundaries. The present investigation examined the influence of brokering on bilinguals' processing of two word collocations with either a literal or a figurative meaning in each language. Proficient Spanish-English bilinguals classified as brokers or non-brokers were asked to judge if adjective+noun phrases presented in each language made sense or not. Phrases with a literal meaning (e.g., *stinging insect*) were interspersed with phrases with a figurative meaning (e.g., *stinging insult*) and non-sensical phrases (e.g., *stinging picnic*). It was hypothesized that plausibility judgments would be facilitated for literal relative to figurative meanings in each language but that experience in language brokering would be associated with a more equivalent pattern of responding across languages. These predictions were confirmed. The findings add to the body of empirical work on individual differences in language processing in bilinguals associated with prior language brokering experience.

## Introduction

Language users make use of linguistic and extralinguistic (e.g., social, cultural) cues to comprehend the intended meaning of an utterance. In some cases the intended meaning follows directly from a computation of the meaning of the constituent elements of the utterance. However, in other cases (as in so-called figurative utterances), the intended meaning of a phrase is not easily derivable from the meaning of its constituent elements but reflects a conventionalized meaning that is culturally shared by users of a language. The present study examined how bilingual language users understand phrases in each of their languages that have a literal meaning vs. a figurative meaning. Further, the study explored the impact of one source of variability among bilinguals on their language processing, namely, the degree to which bilinguals engage in informal translation and thereby have a more interchangeable use of their two languages vs. using each language in different contexts and for different purposes. To date, the notion of complementarity of language use (Grosjean, 2016) has received little empirical attention in the psycholinguistic literature on bilingualism.

The study specifically examined literal vs. figurative phrase plausibility judgments in proficient Spanish-English bilinguals who differed in the degree to which they engaged in informal translation, or language brokering (LB) in early childhood and beyond. Language brokering (LB) is a widespread practice commonly encountered in language contact situations in which immigrant or refugee families call on children or adolescents to serve as linguistic intermediaries for family or community members (Morales and Hanson, 2005). Our study sought to compare the psycholinguistic impact of this practice by comparing bilinguals who are well versed in this practice with bilinguals who have not had to engage in informal translation. To the extent that prolonged experience in informal translation may be expected to predispose dual language users to process the underlying, intended meaning of an utterance, regardless of the language in which it is presented, it was expected that bilinguals with brokering experience would be equally adept at judging the plausibility of phrases in their two languages, regardless of the type of utterance it is (literal or figurative). By contrast, bilinguals without brokering experience might be expected to be more influenced by utterance type and by the language in which the utterance is presented. Before describing our study we first briefly review previous work on the processing of literal and non-literal meaning in users of a single language and multiple languages.

Several studies have examined the processing of utterances in which the intended meaning does not correspond to the literal meaning, as is the case for irony, metaphor, sarcasm, humor, and idiomatic expressions (Gibbs, 1994; Giora, 1997). A particular focus of interest in this body of work is whether there is a processing advantage for literal over non-literal meanings. Three different positions have been outlined. One is the so-called standard pragmatic model (Grice, 1975; Searle, 1979), which posits that a literal meaning of an utterance is computed first and other meanings are considered only if the literal meaning is found not to apply. Challenging this view, the Direct Access Hypothesis (Gibbs, 1994) holds that non-literal meanings may be directly accessed as quickly as literal meanings if sufficient context is available. For example, given the context provided by *surprise party* the figurative meaning of *spill the beans* would be initially primed in the sentence, “Sara spilled the beans about the surprise party,” whereas given a different context, e.g., *stove*, a literal meaning of the phrase would be initially primed in the sentence “Sara spilled the beans all over the stove.” The notion of graded salience, a third position, proposed by Giora (1997; see also Giora et al., 2017), posits that what is initially activated is a *salient default* meaning, which may in some cases be the literal meaning and in others the non-literal meaning.

Until recently (e.g., Vaid, 2006; Heredia and Cieslicka, 2015), studies of figurative language processing were focused largely on single language users. When examining how users of a second or additional language may process utterances with non-literal meanings, additional considerations arise. For example, individuals at the beginning stages of acquiring an additional language might not have sufficient exposure to or familiarity with idiomatic usages, and, thus, might have a propensity to process utterances in that language literally, in comparison to single language users or more advanced second language users (Kecskes, 2006). This position known as the Literal Salience Hypothesis received empirical support in a study by Cieslicka (2006). Using a cross-modal primed lexical decision task, Cieslicka presented participants with English sentences that contained idioms (e.g., *tie the knot*) followed by a target word that was related either to the literal or the idiomatic meaning of the phrase. Lexical decision times to the target word were facilitated when the latter was related to the literal meaning of the phrase, lending support to a Literal Salience Model of idiom processing in second language learners (see also Cieslicka, 2015). Other work with non-native users has similarly shown that they are slowed down in processing figurative meanings, relative to native language users (Siyanova-Chanturia et al., 2011; Carrol and Conklin, 2014, 2015). For example, Siyanova-Chanturia et al. (2011) found that non-native readers processed phrases with a non-literal meaning slower than novel phrases or phrases with a literal meaning. Similarly, Carrol and Conklin (2015) found that, when reading idioms in English, native speakers tended to skip the final word, as it was predictable, while non-native readers did not.

Another important consideration when studying multiple language users is whether idiom processing may be affected by whether or not an idiomatic expression in the target language has a counterpart in the user's other language (e.g., English—e.g., *made of steel* and its counterpart in Spanish, *hecho de acero*). The limited evidence on this issue suggests that, as long as an item has an idiomatic meaning in the primary language, its translation is processed as though it is also idiomatic (Siyanova-Chanturia et al., 2011). Relatedly, it has been shown that expressions that have a shared idiomatic counterpart in both languages of bilinguals are better recalled than those that have an idiomatic meaning in only one language (Pritchett et al., 2016).

Taken together, existing studies, though few in number, suggest that the degree of experience and familiarity with a language, and the presence of idiomatic counterparts in both languages of dual language users, can influence semantic processing of figurative language. What remains to be explored is whether, among individuals with a high level of proficiency in their two languages, differences in how the languages are used, such as in terms of the degree of early language brokering experience, may influence how figurative meaning is processed in each language. This was the aim of the present study, which examined the impact of differences in language brokering experience on how proficient bilinguals comprehend literal and non-literal meaning in their two languages. We next consider previous empirical studies of this individual difference variable.

Experimental investigations of language brokering experience are fairly new and, within this emerging body of work, a few studies have explored the processing of non-literal aspects of language (Vaid et al., 2006, 2015; López and Vaid, 2016).

In an early study, Vaid et al. (2006; see also Vaid et al., 2015, Study 2) hypothesized that LB may facilitate the identification of ambiguity in an utterance. To test this, they devised a humor detection task, reasoning that humor exploits ambiguity. Spanish-English bilinguals, classified on the basis of their prior degree of brokering experience, were asked to judge if visually presented sentences were funny or not. The humorous sentences were taken from actual one-liners, and non-humorous but plausible sentences were created by replacing the final word which conveyed the punchline meaning with another word. Vaid et al. (2006) found that judgments of humorousness (measured by response latencies from the onset of the final word) were faster for brokers than non-brokers, particularly in Spanish, and particularly for humor that relied on extralinguistic knowledge. Thus, brokering experience among Spanish-English bilinguals appears to facilitate comprehension of phrases that exploit ambiguity, particularly for culturally-laden knowledge that is part of the repertoire that brokers make use of.

More recently, López and Vaid (2017) explored Spanish-English brokers' and non-brokers' ability to access idiomatic meaning across languages. A semantic relatedness task was used in which bilinguals were presented with English idioms (e.g., *kick the bucket*) followed by a target word that was either related or not related to the idiom meaning; the target word was in turn presented in either the same language as the idiom (e.g., *death*) or in the other language (e.g., *morir*). The study found that brokers were equally fast and accurate at making semantic relatedness judgments to same language target-idiom pairings as they were to different language pairings. Non-brokers, however, were faster at judging semantic relatedness when the targets were in the same language as the idiom than when they were in the other language.

A study by Tzou et al. (2017), though not on language brokering *per se*, is still relevant. In this study, Mandarin-English speakers who had training in formal translation were compared to untrained bilingual counterparts on a translation verification task involving idioms presented in each language. It was found that the bilinguals with training in translation were equally adept at verifying literal and figurative translations of idioms (and did so equally well in either direction) whereas the untrained bilinguals were faster at verifying literal (verbatim) translations than figurative translations.

Taken together, available studies suggest that translation experience may provide bilinguals with an enhanced ability to detect phrase ambiguity and access the idiomatic meaning of expressions, within and across languages. The present study sought to extend work in this area by directly comparing the relative processing of literal vs. idiomatic meanings of phrases in each language by bilinguals with or without informal translation experience.

In this study, we tested the claim that brokering experience facilitates phrase meaning activation leading to equivalent performance in comprehension of phrases differing in language, regardless of phrase type (literal or non-literal phrase meaning). A phrase plausibility judgment task was developed to test this claim. Phrases were constructed that were either plausible or implausible in their meaning. Plausible phrases were plausible either on the basis of a literal reading or on the basis of a figurative reading. The figurative meaning of a phrase in one language had a shared counterpart in the other language. Stimuli were presented in each language in separate blocks.

In light of previous studies with second language learners that suggest that the literal meaning of a phrase is accessed more readily than its figurative meaning (Cieslicka, 2006), we expected that in the present study as well participants would be better at judging the phrases that had a literal meaning than those that had a non-literal meaning. This pattern was expected to hold across both brokers and non-brokers. We hypothesized that a repercussion of brokering is a more equivalent pattern of response to meaning across the two languages. As such, brokers were expected to show a smaller difference between their two languages in their plausibility judgments relative to that observed in non-brokers, for both literal and non-literal phrases.

## Methods

### Participants

Eighty proficient Spanish-English bilinguals from an introductory Psychology participant pool at a large southwestern university were recruited. Participants were subdivided into non-brokers (*n* = 37) or brokers (*n* = 43) based on their responses on a detailed language background and brokering questionnaire (Vaid, 2012). For brokers, information was coded pertaining to the age at which they started brokering, how often they brokered, what kinds of spoken or written information they brokered, in what settings they engaged in brokering, and for whom they were most likely to broker.

Table 1 summarizes relevant characteristics of the brokers and non-brokers in our sample. There were some demographic differences that would be expected to characterize bilinguals with brokering experience and those with little or no brokering experience. For instance, a majority of non-brokers (83.8%) were born in the U.S. whereas slightly over half of the brokers were U.S.-born (55.8%). More tellingly, the overwhelming majority of parents and grandparents of brokers (over 90%) were born outside the U.S, as compared to about 60% of the parents and grandparents of non-brokers. Not surprisingly, Spanish was the first language of most of the brokers (93.0%) but only about half of the non-brokers (56.8%). Nevertheless, a majority of both groups had acquired their two languages in early childhood (before age 9). However, non-brokers were more likely to have had their elementary schooling in English (72.2%) as compared to that for brokers (46.2%).

**Table 1:** Broker and non-broker language profile.

	**Brokers (*N* = 43)**	**Non-brokers (*N* = 37)**
Percent born in U.S.	55.8%	83.8%
Percent mother born in U.S.	2.3%	35.1%
Percent father born in U.S.	2.3%	37.8%
Percent maternal grandparents born in U.S.	0.0%	27.0%
Percent paternal grandparents born in U.S.	2.3%	29.7%
Spanish as first spoken language	93.0%	56.8%
Percent acquisition of second language before 9 years of age	67.5%	72.9%
Mean composite English proficiency	6.477	6.824
Mean composite Spanish proficiency	6.233	5.514
Percent use of Spanish when speaking with mother	72.1%	27.0%
Percent use of Spanish when speaking with father	62.8%	27.0%
Percent use of Spanish when speaking with grandparents	70.7%	58.3%
Percent use of English in elementary schooling	46.2%	72.2%

In terms of self-reported use of each language, Spanish was reported as the language more commonly used with parents by brokers and English was reported as the language more commonly used with parents by non-brokers. When speaking with grandparents both brokers and non-brokers used Spanish more than English (Spanish: brokers *M* = 70.7% vs. non-brokers *M* = 58.3%). Both brokers and non-brokers reported that they were more effective in communicating in English (Brokers: *M* = 32.6% vs. Non-brokers: *M* = 54.1%) than in Spanish (Brokers: *M* = 20.9% vs. Non-brokers: *M* = 0%).

In terms of relative proficiency in each language, based on self-report, which has been found to be an effective measure of proficiency and one that correlates with objective, behavioral measures of language proficiency (see Flege et al., 2002; Dunn and Fox Tree, 2009), a summary of brokers' and non-brokers' composite proficiency scores and their language background profile is provided in Table 1. Self-reported proficiency on a 7-point scale was obtained for each language in each of four modalities (speaking, reading, writing, and general comprehension) and these were averaged to produce composite ratings. Brokers reported equally high composite proficiency ratings in English (*M* = 6.477; *SD* = 0.721) and Spanish (*M* = 6.233), *t*_(42)_ = 1.588, *p* > 0.05. Non-brokers self-rated their English (*M* = 6.824; *SD* = 0.456) significantly higher than their Spanish (*M* = 5.514; *SD* = 1.233), *t*_(36)_ = 5.989, *p* = 0.0001. When comparing each group on language composite scores, differences emerged between the groups: non-brokers self-rated their English proficiency significantly higher than did brokers, *t*_(78)_ = −2.528, *p* = 0.014, whereas brokers self-rated their Spanish proficiency significantly higher than did non-brokers, *t*_(78)_ = 3.302, *p* = 0.0001 (see Table 1). Although our brokers and non-brokers were not matched in language proficiency in this experiment, this potential confound is addressed in the data analysis and limitations of the study.

### Materials

For each language, stimuli were constructed from a set of 54 triads in both Spanish and English which a given adjective (e.g., “stinging”) was paired with three different nouns (e.g., “insect,” “insult” or “picnic”) such that the resulting two-word phrase either had a plausible literal meaning, a plausible metaphoric meaning, or no plausible meaning (see Supplementary Materials). The figurative phrases used in each language shared a common figurative meaning across both languages (e.g., English—*golden rule* and Spanish—*regla de oro*). Stimuli were developed by a fluent Spanish-English bilingual informant and were pretested on a small sample of other bilinguals to ensure that the figurative meaning of the phrases was discernible. Across participants, all three triads were presented in both languages. While each participant was administered all three stimulus types and was tested in both languages, an individual stimulus was shown in only one form and language to individual participants.

### Procedure

Participants were tested individually in two sessions. During each session, participants performed the plausibility judgments task in one language and the second session was conducted a week later, along with the language background and brokering questionnaire. Language order of the test sessions was counterbalanced.

In a typical trial, participants would see a noun on the computer screen with a line preceding it (for English trials) or following it (for Spanish trials). Participants were then shown an adjective and would have to decide if the resulting phrase made sense or not. They were instructed to make their judgments as quickly and as accurately as possible and to signal their response by pressing the right arrow key to indicate “yes” and the left arrow key to indicate “no.” Timing (in milliseconds) was initiated from the onset of the second word and stopped once the participant made a response. Ten practice trials were given to ensure that participants understood the task, then followed by 27 critical trials with 9 trials per condition (i.e., figurative, literal, and control). This study was carried out in accordance with the recommendations of the Institutional Review Board (IRB) at Texas A&M University (TAMU) with written informed consent from all subjects. All subjects gave written informed consent in accordance with the Declaration of Helsinki. The protocol was approved by IRB at TAMU.

### Data analysis

Response time and accuracy were analyzed. Mean reaction time scores to correct “yes” responses were analyzed in a 2 × 2 × 2 analysis of variance with group (broker vs. non-broker) as a between-subjects factor and phrase language (Spanish vs. English) and phrase type (literal vs. figurative) as within-subjects factors and language proficiency (Spanish language ability and English language ability) as a covariate. Two separate ancovas were conducted, one by-participants (F_1_) and the other by-items (F_2_).

A signal detection analysis was conducted to look at the influence of group, language and phrase type on mean accuracy (based on percent false alarms subtracted from hits). Hits were computed as the degree to which participants correctly responded “yes” to items that were actually plausible. False alarms measure the degree to which participants responded “yes” to items that were actually not plausible. False alarms demonstrate participants' tendency to respond affirmatively. Thus, the mean accuracy analysis reported below examined the degree to which participants correctly said “yes” to items that were actually plausible after subtracting items to which they incorrectly said “yes.” In addition, an analysis of variance on mean percent of misses (failure to say “yes” to items that were plausible) as a function of group, language, and phrase type, was also conducted.

In order to address the potential language proficiency confound, two separate ANCOVAS for reaction time and accuracy rates were analyzed using a subset of participants matched on language proficiency. These analyses resulted in a smaller sample size, but the same group effects were still found. As such, the results reported here are with the larger sample, where we control for language proficiency by running ANCOVAS that included Spanish and English proficiency as covariates.

## Results

### Reaction time analyses

A 2 Phrase Language (English vs. Spanish) × 2 Phrase Type (Literal vs. Figurative) × 2 Group (Broker vs. Non-broker) repeated measures ANCOVA was conducted on mean reaction time of plausibility judgments keeping both Spanish and English language proficiency as covariates. The main effect for phrase language was not significant in the by participants analysis, *F*_1(1, 76)_ = 0.366, *p* > 0.05, η_*p*_^2^ = 0.005, but was significant in the by items analysis; *F*_2(1, 206)_ = 39.206, *p* = 0.0001, η_*p*_^2^ = 0.160. English phrases (*M* = 897.15; *SD* = 251.80) had faster reaction times than Spanish phrases (*M* = 1042.65; *SD* = 291.18). The main effect for phrase type was also significant; *F*_1(1, 79)_ = 5.016, *p* = 0.028, η_*p*_^2^ = 0.060; *F*_2(1, 206)_ = 30.276, *p* = 0.0001, η_*p*_^2^ = 0.128. Literal phrases (*M* = 901.30; *SD* = 186.87) had faster reaction times than figurative phrases (*M* = 1,038.50; *SD* = 0.244.25). There was no main effect of broker status: *F*_1(1, 76)_ = 0.012, *p* > 0.05, η_*p*_^2^ = 0.001; *F*_2(1, 206)_ = 1.339, *p* > 0.05, η_*p*_^2^ = 0.006.

**Figure 1:**
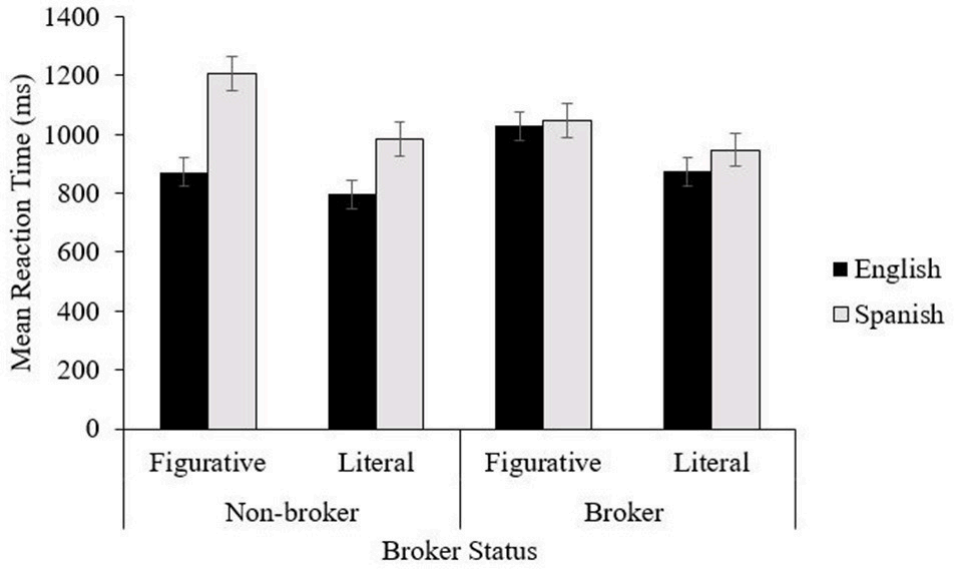
Mean reaction times (ms) across phrase type, phrase language, and group. Error bars represent standard error of means.

The three-way interaction between phrase language, phrase type, and group was also significant, both in the by-participant analysis *F*_1(1, 76)_ = 8.264, *p* = 0.005, η_*p*_^2^ = 0.098 and in the by-item analysis, *F*_2(1, 206)_ = 6.742, *p* < 0.01, η_*p*_^2^ = 032. Follow up *t*-tests demonstrated that for Spanish figurative phrases, brokers (*M* = 1,046.93; *SD* = 260.98) were faster than non-brokers (*M* = 1,207.80; *SD* = 376.16), *t*_(78)_ = −2.246, *p* = 0.028, but for English figurative phrases non-brokers were faster than brokers, (*M* = 871.96; *SD* = 239.85 vs. *M* = 1,027.69, *SD* = 306.24), *t*_(78)_ = 2.502, *p* = 0.014. No group differences were found for English literal phrases, *t*_(78)_ = 1.618, *p* > 0.05, or for Spanish literal phrases, *t*_(78)_ = −0.616, *p* > 0.05 (see Figure 1).

Additional *post-hoc* analysis was conducted with Bonferroni corrections (as *p*') *t*-tests were done looking at the performance of each group by language and phrase type. Brokers were equally fast in responding to English as they were to Spanish, for both literal and for figurative phrases, whereas non-brokers were significantly faster in responding to English than Spanish, for literal, *t*_(36)_ = −4.783, *p*' = 0.0001 and figurative phrases alike, *t*_(36)_ = −5.392, *p*' = 0.0001. Furthermore, whereas both groups responded significantly faster to literal than to figurative phrases in each language, non-brokers were particularly slow in responding to figurative phrases in Spanish (see Figure 1).

### Mean accuracy analyses

Mean accuracy was computed by subtracting false alarms from hits. A 2 Language (Spanish vs. English) × 2 Phrase type (literal vs. figurative) × 2 Broker status (broker vs. non-broker) repeated measures ANCOVA was conducted on mean percent accuracy of plausibility judgments keeping both Spanish and English language proficiency as covariates.

**Figure 2:**
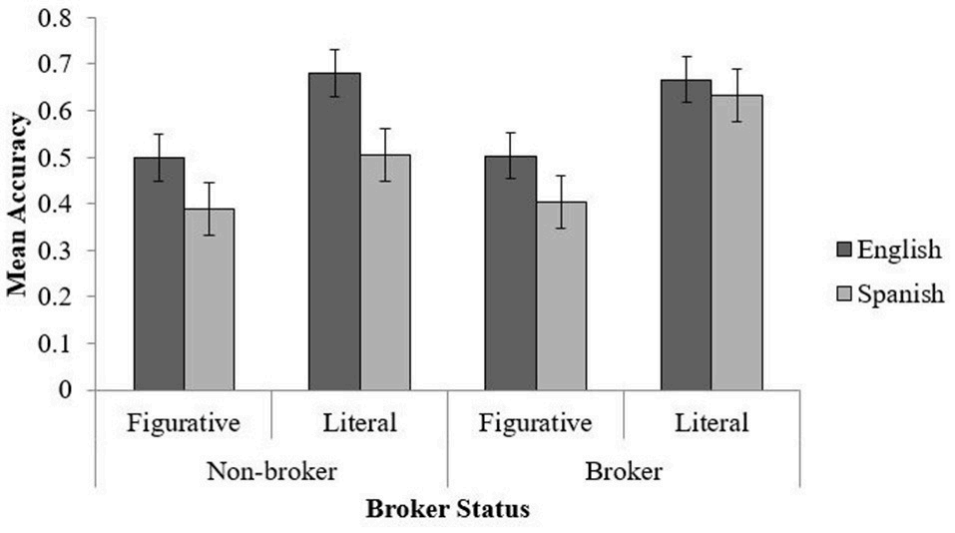
Mean accuracy scores across phrase type, phrase language, and group. Error bars represent standard error of means.

The 3-way interaction between phrase type, phrase language and broker status was significant, *F*_(1, 79)_ = 5.03, *p* = 0.028, η_*p*_^2^ = 0.06. *Post-hoc* analysis was conducted with Bonferroni corrections (as *p*') to follow up the differences between conditions. The *post-hoc* analysis revealed that brokers and non-brokers responded equally accurately for English literal (*M* = 67% vs. 68%), English figurative (*M* = 50% vs. 50%) and Spanish figurative (*M* = 40% vs. 39%) phrases (see Figure 2). However, brokers (*M* = 63%) were almost significantly more accurate for Spanish literal phrases than non-brokers (*M* = 50%); *t*_(81)_ = 2.51, *p*' = 0.09.

Further, for non-brokers there was a significant difference between English literal and figurative phrases, *t*_(36)_ = 5.29, *p*' < 0.012, and between Spanish literal and figurative phrases, *t*_(36)_ = 4.53, *p*' < 0.012. Moreover, when comparing the languages, there was a significant difference between English and Spanish literal phrases [*t*_(36)_ = 3.17, *p*' = 0.024]. However, there was no difference between English and Spanish figurative phrases [*t*_(36)_ = 2.06, *p*' = 0.27].

Similar to non-brokers, brokers judged English literal phrases more accurately than figurative phrases [*t*_(45)_ = 6.27, *p*' < 0.012] and Spanish literal phrases than figurative phrases [*t*_(45)_ = 9.17, *p*' < 0.012]. On the other hand, differently than non-brokers, the difference between English and Spanish literal phrases was not significant, *t*_(45)_ = 0.76, *p*' = 1 further between English and Spanish figurative phrases for brokers was not significant, *t*_(45)_ = 1.96, *p*' = 0.28, and they did not show any difference. No other main effects and interactions were significant.

#### Mean missed analyses

A 2 Language (Spanish vs. English) × 2 Phrase type (literal vs. figurative) × 2 Broker status (broker vs. non-broker) repeated measures ANCOVA was conducted on misses, that is, failing to judge something that was plausible as being plausible keeping both Spanish and English language proficiency as covariates.

The phrase type by phrase language by broker status interaction was significant, *F*_(1, 79)_ = 5.03, *p* = 0.02, η_*p*_^2^ = 0.06. There was no difference found between brokers and non-brokers in English literal [*t*_(79)_ = 0.57, *p*' = 1], English figurative [*t*_(79)_ = 0.09, *p*' = 1], Spanish literal [*t*_(79)_ = 1.83, *p*' = 0.42] and Spanish figurative [*t*_(79)_ = 1.52, *p*' = 0.65] phrases.

Within non-brokers English figurative phrases were missed more than literal phrases, *t*_(36)_ = 5.29, *p*' < 0.012. Similarly Spanish figurative phrases were missed more than literal phrases, *t*_(36)_ = 4.53, *p*' < 0.012. When comparing languages within same phrase type, Spanish literal phrases were missed more than English literal phrases, *t*_(36)_ = 3.33, *p*' = 0.016. However, there was no difference between Spanish and English figurative phrases, *t*_(36)_ = 0.95, *p*' = 1.

Within brokers, phrase type comparisons within the same language were similar to non-brokers. English figurative phrases were missed more than English literal phrases, *t*_(45)_ = 6.27, *p*' < 0.012. Spanish figurative phrases were missed more than literal phrases, *t*_(45)_ = 0.9.17, *p*' < 0.012. Brokers did not miss Spanish figurative phrases than English figurative phrases, *t*_(45)_ = 2.41, *p*' = 0.14. Further they did not show any difference between English and Spanish literal phrases, *t*_(45)_ = 1.03, *p*' = 1. No other main effects and interactions were significant.

## Discussion

We had hypothesized that judgments of the plausibility of a phrase would be facilitated when the phrase's meaning is literal, that is, easily derivable from the meaning of its constituent elements, than when the meaning is non-literal and based on a conventional, idiomatic meaning assigned to the phrase. Our findings support a robust effect of phrase type in making plausibility judgments. For phrases that were judged accurately, responses were significantly faster for literal than for figurative phrase meanings, in both groups and across both languages. This suggests that, at least when literal and figurative phrases are randomly intermixed, phrases that have a literal meaning are processed more easily by bilinguals than those with a figurative meaning. Although, the present study presented phrases with figurative or literal meaning without context to two groups of proficient bilinguals, these bilinguals were still faster and more accurate for phrases with literal meaning compared to those with figurative meaning, regardless of language of presentation. As such, our results extend the scope of the Literal Salience Model of Cieslicka (2015) to proficient bilinguals.

In addition, we found a strong language effect, with plausibility judgments being faster and more accurate for English than for Spanish phrases. However, the language effect interacted with broker status, indicating that this language dominance effect mainly characterized non-brokers. The superiority of English over Spanish noted for the non-brokers may have been contributed in part by the fact that a majority of non-brokers received their early schooling in English. Of particular relevance is the finding that brokers responded in a similar manner to phrases presented in Spanish as they did to phrases presented in English, regardless of phrase type. This finding supports the notion that a byproduct of prolonged experience in informal translation is to make both languages more readily accessible.

The pattern of results from the accuracy analysis is consistent with that from the reaction time analysis in showing that participants recognized phrases with a literal meaning more accurately than those with a figurative meaning, and English phrases more accurately than Spanish phrases. However, as hypothesized, brokers showed more comparable performance across their languages whereas non-brokers performed better in English than in Spanish. The finding of higher accuracy rates for literal phrases (and correspondingly, more misses for figurative than literal phrases) suggests that, when presented with figurative language without context, bilinguals are more readily prepared to judge a literal meaning as plausible than a plausible figurative meaning. As noted above, this effect is in line with the literal salience hypothesis (Cieslicka, 2006) and standard pragmatic model (Gibbs, 1994; Giora, 1997). The former posits that second language users first process the literal phrase of an idiom prior to a figurative meaning, while the latter posits that the literal meaning of figurative phrases holds precedence over the figurative. Our study sample included highly proficient Spanish English bilinguals, not second language learners. The fact that they still were more accurate in judging phrases with a literal meaning than a figurative meaning is noteworthy. However, it is important to remember that our study found these effects for phrases that were presented without any context. The possibility here is then that when phrases presented without context, the literal meaning advantage may be heightened. Therefore, in the future it would be important to view effects of biasing context embedded with potential literal or figurative phrases.

In summary, the present study found support for a theorized effect of language brokering experience in that brokers showed a more equivalent level of responding in their two languages than did non-brokers. However, both brokers and non-brokers alike were faster and more accurate in responding to phrases with a literal meaning than those with a figurative meaning, at least within the circumstances in which these phrases were presented in the present study that is, randomly intermixed. It is possible that if the literal and non-literal phrases had been presented in a blocked fashion, the size of the literal meaning advantage might have been reduced. This possibility remains to be investigated.

### Limitations

One potential limitation in this study is that phrases were presented in the absence of a constraining context that could prime either the literal or the figurative meaning. In future work, it would be interesting to add contextual cues. Under those conditions, it would be interesting to study if context would facilitate the processing of the figurative meaning to the same extent for brokers and non-brokers, or whether one group would show a greater benefit.

An additional factor that limits the generalizability of the study is that the figurative phrases used all involved a shared meaning in both languages. Thus, it remains unknown how the groups may have responded when a phrase has a figurative meaning in only one language. Relatedly, the languages themselves in the present study were relatively close in their structure. It would be important in future work to test language pairs that are typologically more distinct to see if that would make a difference in the present task.

Finally, the variable of language brokering status was conceptualized in the present study as a dichotomous variable. It may be informative to treat it as a graded variable, e.g., to look at whether there is a certain frequency of brokering experience above which differences emerge. Other dimensions of brokering experience besides frequency (e.g., use of brokering for everyday interaction vs. for specialized, technical translation) may be worth exploring in future work to provide a more comprehensive and nuanced investigation of what aspects of brokering experience affect language processing. Additionally, language proficiency among our brokers and non-brokers varied, which could have been a potential confound, this difference did not significantly influence our results. As such, future work on language brokering will need to control and account for potential variances in language proficiency among brokers and non-brokers.

### Implications

The present findings extend and add to previous experimental studies of language brokering (Vaid et al., 2006, 2015; López and Vaid, 2016, 2017). Bilinguals with extensive prior language brokering experience behave differently from those without it on a task involving the judgment of phrase plausibility in which plausibility was varied in two ways. Whereas brokers and non-brokers alike were faster and better at judging plausibility in each language when it was based on the literal meaning of the phrase than on its figurative meaning, brokers were somewhat better than non-brokers on this task, and performed more comparably across their two languages on all measures (reaction time, mean accuracy). More generally, our findings suggest that brokers are more cautious and deliberate when judging if a phrase is meaningful, regardless of the language as well as the type of meaning. Also, comparable to López and Vaid (2017), it appears that for brokers, performance on their two languages is more similar whereas for non-brokers there is more of an effect of language.

With regard to the figurative language literature in multiple language users, the present investigation shows that a byproduct of prolonged experience in informal translation is to make both languages more readily accessible. Our findings extend the study of figurative language processing beyond the comparison of non-native speakers and native-speakers (Siyanova-Chanturia et al., 2011; Carrol and Conklin, 2014) to considering differences that may exist between advanced multiple language users. Importantly, our study adds to the growing number of studies that document metalinguistic repercussions of early language practices such as language brokering. Valdés (2003) and Orellana (2009) noted that child or adolescent interpreters show sophisticated language and pragmatic skills well beyond what would be expected for their age. Our study demonstrates that language brokering experience may have lasting effects on semantic processing even into young adulthood.

This idea of individual differences and its effects on language processing has also been suggested by various researchers (Grosjean and Li, 2013; Baum and Titone, 2014). Future research can benefit from taking into account how systematic differences in language experience such as language brokering may have lasting effects on cognitive and linguistic processing. By moving away from the bilingual and monolingual dichotomy, bilingualism researchers will be able to investigate how various language practices ultimately affect how bilinguals interact, comprehend and use their languages.

## Author contributions

All four authors (BL, JV, ST, and CR) contributed to the manuscript in terms of conception and design of experimental stimuli and procedure (BL, CR, JV), implementation of experimental protocols, data collection and analysis (BL, ST), and editing of drafts (BL, ST, JV).

### Conflict of interest statement

The authors declare that the research was conducted in the absence of any commercial or financial relationships that could be construed as a potential conflict of interest.
