# Supplementary material for: Bilinguals' Plausibility Judgments for Phrases with a Literal vs. Non-literal Meaning: The Influence of Language Brokering Experience
Source: Front Psychol. 2017 Sep 25;8:1661. doi: 10.3389/fpsyg.2017.01661 (PMC5626980; doi:10.3389/fpsyg.2017.01661)
Supplement: Supplementary file 1 [file DataSheet1.docx]

# Appendix A: Experimental Stimuli

**English Phrases:**

Rich Businessman Rich Chocolate Rich Parachute

Golden Ring Golden Rule Golden Air

Round Box Round Sum Round Breath

Sharp Scissors Sharp Eyesight Sharp Telephone

Flimsy Frame Flimsy Excuse Flimsy Hole

Greasy Counter Greasy Smile Greasy Music

High Mountains High Expectations High Smell

Sticky Confection Sticky Situation Sticky Restaurant

Refined Oil Refined Lady Refined Bill

Cold Wind Cold Aura Cold Diary

Transparent Plastic Transparent Accounts Transparent Festival

Black Shoes Black Mood Black Force

Sweet Taste Sweet Girl Sweet Itch

Green Grass Green Envy Green Sound

Open Door Open Heart Open Nail

Empty Carton Empty Words Empty Cinder

Thorny Cactus Thorny Problem Thorny Lotion

Closed Store Closed Mind Closed Smoke

Rigid Muscles Rigid Thinking Rigid Printer

Fine Powder Fine Clothes Fine Shout

Sour Cream Sour Manner Sour Shelf

Crooked Line Crooked Deal Crooked Calm

Pure Water Pure Soul Pure Bump

Brilliant Diamond Brilliant Scientist Brilliant Microphone

Dirty Dishes Dirty Jokes Dirty Pounds

Tight Pullover Tight Schedule Tight Surgeon

Loose Shirt Loose Tongue Loose Maroon

Red Plum Red Rage Red Wish

Silky Hair Silky Voice Silky Roof

Juicy Orange Juicy News Juicy Pouch

Warm Climate Warm Greeting Warm Ticket

Soft Scarf Soft Policy Soft Mystery

White Cloud White Noise White Degree

Loaded Shotgun Loaded Statement Loaded Temperature

Rough Surface Rough Comment Rough Pudding

Frozen Hands Frozen Time Frozen Pain

Steel Gate Steel Nerves Steel Snack

Flexible Legs Flexible Answer Flexible Tickle

Hanging Clothes Hanging Matter Hanging Floor

Absent Student Absent Stare Absent Height

Delicious Dessert Delicious Laughter Delicious Spandex

Blind Boy Blind Love Blind Wood

Cancerous Tissue Cancerous Hatred Cancerous Cutlet

Muddy River Muddy Issue Muddy Lamp

Clean Washcloth Clean Conscience Clean Sympathy

Stained Overalls Stained Reputation Stained Reaction

Wounded Thigh Wounded Pride Wounded House

Broken Bones Broken Spirit Broken Syrup

Rotten Banana Rotten Feeling Rotten Concrete

Deep Lagoon Deep Reflection Deep Physique

Bitter Olives Bitter Memory Bitter Bedroom

Hungry Mouth Hungry Ears Hungry Screen

Dead Person Dead Center Dead Flight

Deadly Stab Deadly Charm Deadly Fiction

**Spanish Phrases:**

Empresario Rico Chocolate Rico Paracaídas Rico

Anillo De Oro Regla De Oro Aire De Oro

Caja Redondo/a Suma Redondo/a Ruido Redondo/a

Tijeras Agudo/a (s) Vista Agudo/a (s) Teléfono Agudo/a (s)

Armazón Debíl Excusa Debíl Hueco Debíl

Mostrador Grasosa Sonrisa Grasosa Música Grasosa

Montañas Alto/a (s) Expectaciónes Alto/a (s) Olor Alto/a (s)

Confección Pegajosa Situación Pegajosa Restaurante Pegajosa

Aceite Refinado/a Señora Refinado/a Cuenta Refinado/a

Viento Frío/a Aura Frío/a Diario Frío/a

Plástico Transparente(s) Cuentos Transparente(s) Festival Transparente(s)

Zapatos Negro/a (s) Humor Negro/a (s) Fuerza Negro/a (s)

Sabor Dulce Niña Dulce Picor Dulce

Hierba Verde Envidia Verde Sonido Verde

Puerta Abierto/a Corazón Abierto/a Clavo Abierto/a

Cartón Vacío/a (s) Palabras Vacío/a (s) Ceniza Vacío/a (s)

Cactus Espinoso/a Problema Espinoso/a Loción Espinoso/a

Tienda Cerrado/a Mente Cerrado/a Humo Cerrado/a

Músculos Rígido/a (s) Pensar Rígido/a (s) Impresora Rígido/a (s)

Polvo Fino/a Ropa Fino/a Grito Fino/a

Crema Agrio Manera Agrio Estante Agrio

Línea Chueco/a Trato Chueco/a Calma Chueco/a

Agua Puro/a Alma Puro/a Golpe Puro/a

Diamante Brillante Científico Brillante Micrófono Brillante

Platos Sucio/a (s) Chistes Sucio/a (s) Libras Sucio/a (s)

Abrigo Apretado/a Horario Apretado/a Cirujano Apretado/a

Camisa Suelto/a (s) Lengua Suelto/a (s) Granate Suelto/a (s)

Ciruela Rojo/a Furia Rojo/a Deseo Rojo/a

Pelo Sedoso/a Voz Sedoso/a Techo Sedoso/a

Naranja Jugoso/a Noticia Jugoso/a Bolsillo Jugoso/a

Clima Cálido Saludo Cálido Billete Cálido

Bufanda Suave Política Suave Misterio Suave

Nube Blanco/a Ruído Blanco/a Grado Blanco/a

Escopeta Cargado/a Declaración Cargado/a Temperatura Cargado/a

Superficie Áspero/a Comentario Áspero/a Gelatina Áspero/a

Manos Congelado (s) Tiempo Congelado (s) Dolor Congelado (s)

Portillo De Hierro Nervios De Hierro Bocado De Hierro

Piernas Flexible (s) Respuesta Flexible (s) Cosquilla Flexible (s)

Ropa Pendiente Tema Pendiente Piso Pendiente

Estudiante Ausente Mirada Ausente Altura Ausente

Postre Delicioso/a Risa Delicioso/a Spandex Delicioso/a

Niño Ciego/a Amor Ciego/a Madera Ciego/a

Tejido Canceroso/a Odio Canceroso/a Chuleta Canceroso/a

Arroyo Turbio Asunto Turbio Lámpara Turbio

Toallita Limpia Conciencia Limpia Misericordia Limpia

Sobretodo Manchado/a Reputación Manchado/a Reacción Manchado/a

Muslo Herido/a Orgullo Herido/a Casita Herido/a

Huesos Roto/a (s) Espíritu Roto/a (s) Jarabe Roto/a (s)

Banana Podrido Sentimiento Podrido Concreto Podrido

Laguna Profundo Reflección Profundo Físico Profundo

Aceituna Amargo/a Recuerdo Amargo/a Habitación Amargo/a

Boca Hambrienta (s) Orejas Hambrienta (s) Pantalla Hambrienta (s)

Persona Muerto/a Punto Muerto/a Vuelo Muerto/a

Puñalada Fatal Encanto Fatal Ficción Fatal
